# Using historical tropical cyclone climate datasets to examine wind speed recurrence for coastal Australia

**DOI:** 10.1038/s41598-022-14842-2

**Published:** 2022-07-08

**Authors:** S. S. Bell, A. J. Dowdy, H. A. Ramsay, S. S. Chand, C-H Su, H. Ye

**Affiliations:** 1 Bureau of Meteorology, Melbourne, Australia; 2 CSIRO Oceans and Atmosphere, Aspendale, Australia; 3 School of Engineering, IT and Physical Sciences, Federation University, Ballarat, Australia

**Keywords:** Atmospheric science, Climate change, Engineering

## Abstract

Likelihood estimates of extreme winds, including those from tropical cyclones (TCs) at certain locations are used to inform wind load standards for structural design. Here, wind speed average recurrence intervals (ARIs) determined from TC climate data dating back to the 1970s in two quantile–quantile adjusted reanalysis datasets (ERA5 and BARRA [1990]), and best-track observations for context, were compared with Standardized ARIs (AS/NZS) across seven tropical and two subtropical Australian inland coastal regions. The novelty of this work lies in determining TC-wind speed ARIs from a range of datasets that are not typically used to evaluate this metric. Inherent differences between the data used to determine the Standard ARIs (large sample size allow for larger extrapolations; GEV function) and TC data ARIs (smaller sample size and less certain data; the more asymptotic Lognormal/Weibull functions are used) led to the use of different extreme value functions. Results indicated that although these are two distinct ways of determining design wind speeds, when they are considered equivalent, there was a moderate reproduction of the ARI curves with respect to the Standard in both reanalysis datasets, suggesting that similar analyses using climate model products can provide useful information on these types of metrics with some caveats. Trends in TC wind strength affecting coastal Australia were also analyzed, indicating a potential slight downtrend in tropical West coast TC wind strength and slight uptrend for tropical East coast TC wind strength, noting considerable uncertainty given the short time period and limitations of data quality including over longer time periods. Such trends are not only limited to the relationship between TC intensity and anthropogenic warming, but also to regional changes in TC frequency and track direction. This could lead to significant trends emerging in regional Australian TC wind gust strength before several decades of warming have occurred. It is hoped that climate models can provide both longer-term and a more homogenous base for these types of evaluations and subsequent projections with respect to climate change simulations.

## Introduction

Extreme wind speeds induced by tropical cyclones (TCs) can have considerable impacts on coastal communities and infrastructure situated in tropical Australia. In light of anthropogenic-induced climate change, it is important to investigate the potential evolution of TC-wind hazards for the benefit of future planning and disaster risk management in Australia.

The expected average recurrence interval (ARI) of extreme wind speeds is a metric widely used by engineers and climate scientists alike to gauge the wind hazard for specific regions. In Australia, this is described by the “structural design actions” for winds^1^ herein referred to as “the Standard”. Different regions of Australia are assigned various designations (A, B, C or D, in ascending order of strength) based on the historical wind strength (ARI) observed in that region. For example, Australia’s tropical and subtropical coastlines have much higher designations (B–D) than the southern and inland regions (A) due to the increased exposure to TCs. Historically, appropriate regional wind speed designations have been determined by weather station surface anemometer measurements (corrected for terrain, topography and gust duration) and to a lesser extent damage surveys from past events (including in some cases back calculations of maximum wind speeds from failed simple structures such as road signs) and these methods remain very useful.

In the latest edition of the AS/NZS structural design Standard (1170.2:2021), for the first time, a climate change multiplier (1.05) was included as mandatory to consider in some regions of Australia. This is consistent with a broad review and synthesis of available lines of evidence where it is generally accepted that global TC intensity will increase with projected future warming on the order of 3–5% with an additional 2 °C warming^2,3^ For local context, the East coast of Australia has experienced a recent influx of severe Australian Category 4/5 TCs^4^ (Table 1) while the West coast of Australia has experienced fewer Category 4/5 TCs recently^5^. This leads to the question of what role can climate model experiments play in informing us of likely future changes in fine-scale metrics such as wind speed ARIs that have traditionally come from observed ground truth data. Climate models, despite having their own limitations such as biases and inaccurate representations of some small- and large-scale physical processes, still provide homogenous simulations that can be useful for examining the potential influence of climate change now and into the future, as a complementary line of evidence to observations-based data (which also have limitations including due to evolving operational practices for wind observations in Australia with a relatively short time period of high-quality data being available).

**Table 1:** The Australian TC wind scale as defined by wind gust speed (3-s).

Category	Gust speed
1	24.5–34 m s^−1^
2	34–45.5 m s^−1^
3	45.5–61.6 m s^−1^
4	61.6–77.4 m s^−1^
5	> 77.4 m s^−1^

It is timely that with the recent release of new, finer resolution reanalysis products, including the Bureau of Meteorology Atmospheric high-resolution Regional Reanalysis for Australia (BARRA^6^) and the global ERA5 reanalysis^7^ produced by the ECMWF; the feasibility of determining wind speed ARIs in climate model type products can be tested. This can be achieved by direct comparison with the ground truth determined wind speed ARIs from AS/NZS 1170.2. The two reanalysis datasets can also provide additional lines of evidence in relation to current historical TC trends and extreme wind speeds affecting Australia.

The simulation of peak wind gust speeds from TCs in reanalysis products poses many challenges, and the products used here do not completely resolve the absolute wind gust speeds of the more intense TCs^2,8^. However, by calibrating the reanalysis data to the observations, such as from Automatic Weather Stations (AWS) and from the best track estimates of TC wind speeds^9–11^ useful information on absolute gust speeds in reanalysis data can be obtained, though with some obvious limitations^12^. That is, the ARIs calculated here, and similar analyses could potentially help with the evolution of the structural AS/NZS wind speed Standard going forward. For example, to assist with defining the weakening rates of TCs in inland locations (where station coverage is more limited) as well as ensuring that existing regional wind designations are correct, including the projected impacts of climate change. Secondly, these analyses can serve as an additional line of evidence contributing to a more comprehensive understanding of potential long-term changes in this metric and in the risk of TC-wind hazards for coastal Australia. In particular, several data sets are used here for the first time to examine TCs and associated extreme wind speeds in these regions, thereby providing new insight on this important topic, which is intended to help provide useful guidance complementary to existing information.

**Figure 1:**
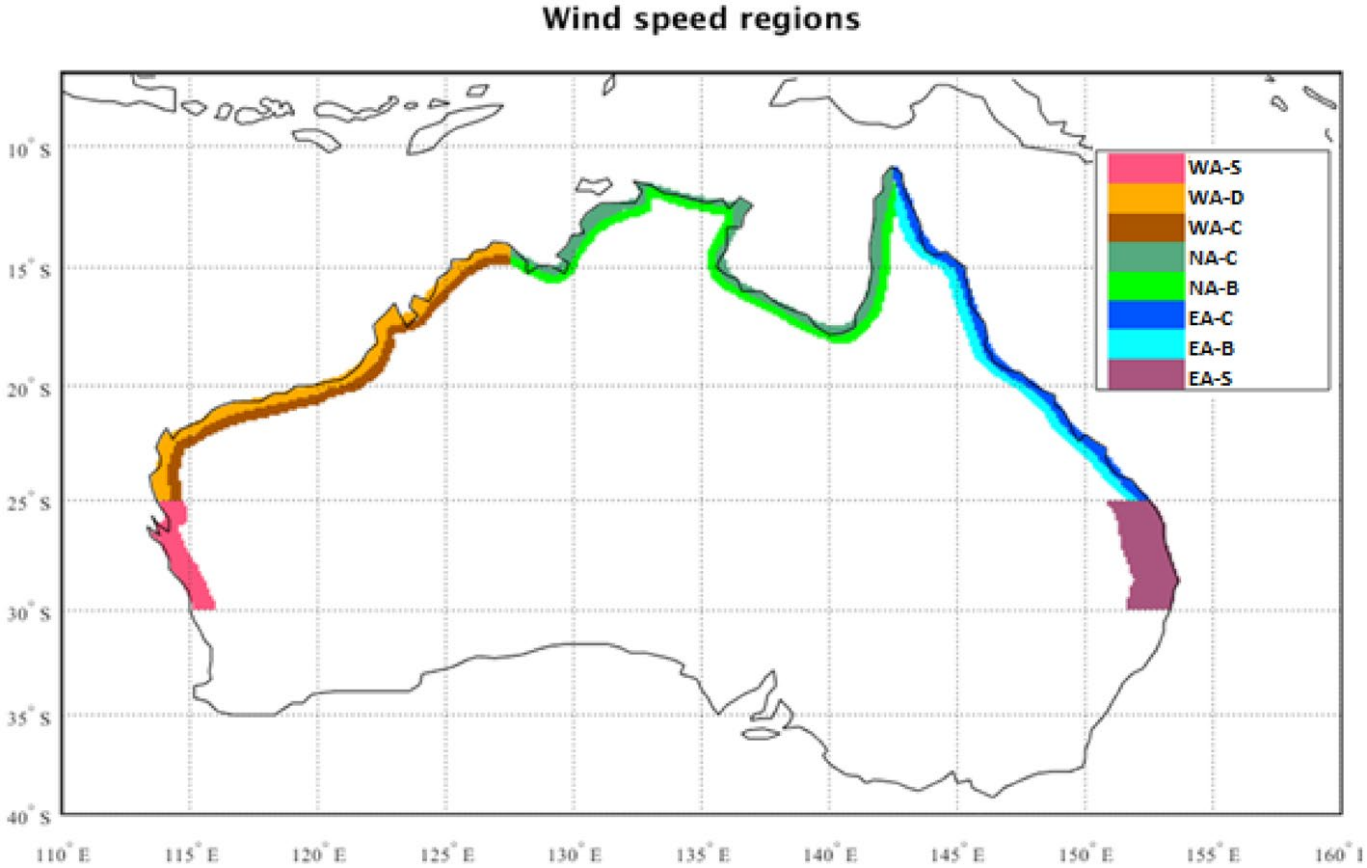
Wind speed regions used in this study. These regions have been designated by the historical wind strength the region has experienced (AS/NZS 1170.2:2021). Considering the strongest designation in each of our regions, in descending order of strength these correspond to: Region D (WA-D), Region C (WA-C, WA-S, NA-C and EA-C) and Region B (NA-B, EA-B and EA-S).

Here, we evaluate ARIs of TC-related daily-maximum wind gusts over eight Australian coastal regions (Fig. 1). These regions closely correspond with those designated by AS/NZS 1170.2:2021, allowing for a comparison of these wind speeds to the Standard. Two subtropical regions on either side of the Australian continent, where weakening TCs and extratropical cyclones (ETCs) can sometimes occur, are additionally examined. Examination of these two regions may provide some guidance on a key scientific question around the potential poleward movement of TCs and their wind hazards. The following section provides details on the Data and Methods used. Results are shown in “Results” section , and a Summary and Conclusion is provided in “Discussion” section.

## Data and methods

### Data

#### Best track

The Bureau of Meteorology (BoM) TC dataset (available online at http://www.bom.gov.au/cyclone/tropical-cyclone-knowledge-centre/databases/) includes historical TCs that have occurred in the Australian region (from 90 to 160 degrees East). Since the introduction of operational satellite technology in the late 1960s for the Australian region, the historical records of TCs (analyzed here over the period 1971 to 2021), have included best track sustained wind speed, wind gust estimates, and TC position and time information (with a degree of uncertainty^13^). We note that some TCs in the most recent two years of the dataset are considered provisional, meaning they have not yet undergone formal best track review; and that several TCs in the early 1970s do not have wind speed information available; further noting that geostationary satellite imagery only became available in 1978^15^.

TC wind speed estimates in the best track data have been most widely determined by the satellite-based Dvorak technique^9,10,14^, and in Australia this was first used for TC intensity estimation in 1972^16^. This technique involves the subjective assignment of Dvorak numbers to satellite imagery of TCs to give an indication of the wind speed. However, especially more recently, other tools and techniques have been used by operational forecast centres and meteorological agencies to estimate TC intensities. Such tools and techniques, which include scatterometers, AWS, as well as the Advanced Dvorak technique (ADT), and the SATellite CONsensus intensity approach (SATCON), can influence the final Dvorak number estimate (and therefore the intensity) that is eventually recorded in the best track data^11^. These new tools and techniques make the recent (~ post 2003) best-track record more accurate^16^, but also embeds a degree of inhomogeneity in the dataset when compared with the earlier periods. As such, numerous studies have recommended data from more recent periods for evaluations of TC intensity estimates^17^. For example^18^, recommends data from 1980/81 onwards for TC intensity analysis, whereas others are more conservative: recommending data from 1990 onwards^19^ and from 2003 onwards^20^. In our case, we have used data dating back to 1971 which was a subjective take of where there were enough TCs with intensity information for meaningful analysis, however we have placed emphasis on the period from 1990 onwards.

A re-evaluation of TCs in the Australian region^20,21^ provides revised wind speed (V_max_) estimates in many instances compared to those in the best-track (BoM) TC dataset over the period 1981–2016, by for example, using ADT to remove subjectivity from the simple Dvorak technique. In addition, there are some cases in the best track dataset where only a TC’s sustained wind speed (i.e., 10-min average value) was recorded in the best track TC dataset (i.e., no wind gust conversion based on an open terrain estimate is provided). Here, 3-s wind gusts were obtained by multiplying the wind speed value by a gust factor (GF) of 1.4. GFs can vary due to several factors^22^, including changes in surface roughness (where complex terrain requires a higher GF than that of the smooth open ocean^23^). Similarly, there are also various modification factors that determine the actual wind speed a specific site will experience. These factors include the directional, shielding, terrain and topographic multipliers (e.g., AS/NZS 1170.2). Due to the broad scale of the analyses undertaken here, a GF of 1.4 was used when necessary, noting a similar value was used in most best track derivations of wind gust speed (that vary depending on terrain).

As discussed in the previous paragraph, there are three options to use for representing best track wind gust values. We give a preference to the Courtney and Burton^21^ adjusted V_max_ (10-min sustained) wind speeds, where available over the period 1980–2016, ahead of the best-track wind speeds such that the final TC wind gust speed values assigned to TC observations are in the following order:

- Adjusted V_max_ × 1.4 (Courtney and Burton reanalysis^21^)
- Best track wind gust speed estimate
- Best track V_max_ × 1.4

#### ERA5

The latest global reanalysis produced by the European Centre for Medium-Range Weather Forecasts (ECMWF), ERA5^7^, is available at the horizontal resolution of 31 km. Daily maximum wind gusts (averaged over 3-s at 10 m height) were calculated from hourly maximum values over the period spanning 1979 to June 2021 (available online at https://cds.climate.copernicus.eu/cdsapp#!/dataset/reanalysis-era5-single-levels?tab=form). Note that the effects of convection are only included in the wind gust parameterization from October 2008 onwards which embeds a degree of non-homogeneity in the data as is the case in general for data based on observations.

**Figure 2:**
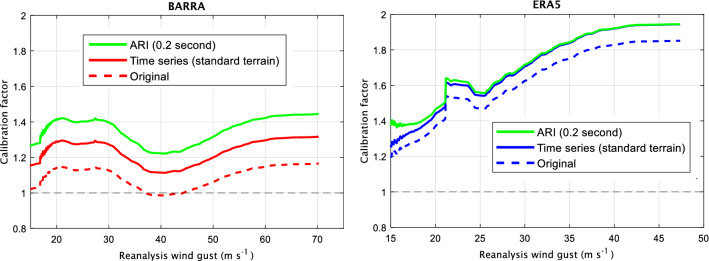
Smoothed calibration factors that are applied to reanalysis wind gusts to make them more consistent with observation wind gust values. A calibration factor of 1 indicates no calibration is required.

As the typical radius of maximum wind (RMW) of an intense TC is around 35 km, there is little chance for capturing the true maximum in model output such as ERA5 and so, for comparison with the Standard, the ERA5 wind speeds are calibrated to observational data (mostly derived over the ocean) using quantile–quantile scaling (Fig. 2). These winds are then further converted to standard terrain conditions (i.e., over land) by a factor of 1.05 ^12^. For ARI calculations, the daily-maximum wind gusts are additionally converted to a 0.2 s averaging period (1.1), that linearly decreases in strength for the distribution tail to avoid exacerbating these already large absolute values (due to the relative coarseness of the ERA5 data).

#### BARRA

BARRA is a 12 km horizontal grid spacing reanalysis product covering the Australian region, (65°E–163°W, 20°N–60°S). This reanalysis includes assimilation of central pressure observations from the International Best Track Archive for Climate Stewardship (IBTrACS^24^). Maximum hourly surface (10 m) wind gust speeds were acquired and used to calculate daily maximum wind gust values over the available period spanning 1990 to February 2019.

There are several known issues in the BARRA data^25^, including grid-sized storms and anomalous wind gust spikes that can produce exceedingly large wind gust values at single isolated grid-points, particularly over northern Australia and near coastlines. To circumvent this issue, the hourly maximum grid cell for a specific region is discarded and replaced by the second-maximum value. The hour-on-hour increase in maximum wind gust speed is also limited to 20 ms^−1^ for the final calculation of daily-maximum wind gust speeds. In cases where there is no grid-point storm or extreme gust spike present, this method usually results in a negligible reduction (< 1 ms^−1^) in the value of the maximum wind gust speed.

As was done for the ERA5 data, the BARRA reanalysis data are calibrated using quantile–quantile scaling to match the observational data while preserving the original distribution of the data (Fig. 2). Slightly different to the ERA5 data, the entire BARRA distribution is converted to an averaging period of 0.2 s (1.1) and a larger value is used^1,12^ (1.13) to convert to standard terrain conditions (different stages of calibration are shown in Fig. 2). As compared to the calibration of ERA5 data, the finer resolution BARRA data required smaller calibration factors (Fig. 2) such that this approach provides a suitable means of reducing limitations of the reanalysis products while producing calibrated data that are more consistent with the observations in absolute terms.

### Methods

#### Wind speed regions

Eight wind speed regions (Fig. 1) are defined here, where the tropical regions are denoted by their AZ/NZS 1170.2 designation “-B”, “-C” or “-D” suffix. The two subtropical regions are simply denoted by the “-S” suffix. The strongest designation in each region corresponds to the regions used here uniformly with exception of the WA-S region which lies only partly under the Region-C designation (between 25 and 27°S).

#### TC-related wind gusts

Measures of wind gust maxima are confined to the eight wind speed regions as shown in Fig. 1. Daily maximum wind gusts for these regions are considered to be “TC-related” if there is a TC track position either inside the region, or its centre is within a 4° radius on the same day as the daily-maximum wind gust. Such “days” that are determined to be TC-related are referred to as “TC days”. The TC data is defined from the BoM TC dataset (best track) and extra-tropical transition days as flagged in the database were not removed from the analysis.

#### Detection of ETC tracks and ETC-related wind gusts

For the two subtropical regions denoted with the suffix “-S” in Fig. 1, extratropical cyclone (ETC) days are also considered, in addition to TC days (i.e., days on which ETCs occur inside or within 4° radius of a given region). The cyclone tracking scheme of Murray and Simmonds^26^ with refinements^27^ was used to detect their occurrence in ERA5 data over the full time period and applied to both ERA5 and BARRA wind gust data. This scheme has been applied previously in reanalysis data to produce ETC tracks^28^. The number of ETCs was not tuned to any existing climatological records, and so lower intensity circulations can remain in our definition of “ETC days”. Detected ETCs were differentiated from other storms, such as detected TCs, by having a genesis location poleward of 23°S.

#### Extreme value modelling, ARIs and peaks

The method of peak-over-threshold was used to model extreme daily-maximum cyclone-related wind gust data, where the threshold was arbitrarily chosen to produce a total average of ~ 3 peaks per year. In cases where there are insufficient peaks for a set of data/region (e.g., subtropical regions and best track data) the data is resampled with replacement to achieve the desired number of peaks. This procedure is repeated several times to ensure the most extreme values are not oversampled. Confidence bounds for ARI curves are derived by removing (lower bound) and duplicating (upper bound) the most extreme values (top 5%).

The lognormal and Weibull functions best modelled the tails of our wind gust distributions in comparison to other functions (such as GEV and Gumbel; not shown). It should be noted that the Standard curves are derived using GEV, while curves derived from our data are more asymptotic (based on lognormal and Weibull shapes). The parameter estimation method of maximum likelihood was preferred although others could have been used (e.g., L-moments^29^). In cases where the lognormal function did not provide a good fit to our data (mostly for cases with tail-heavy distributions on the West coast and for best track data), the Weibull distribution was used instead.

## Results

### Time series

**Figure 3:**
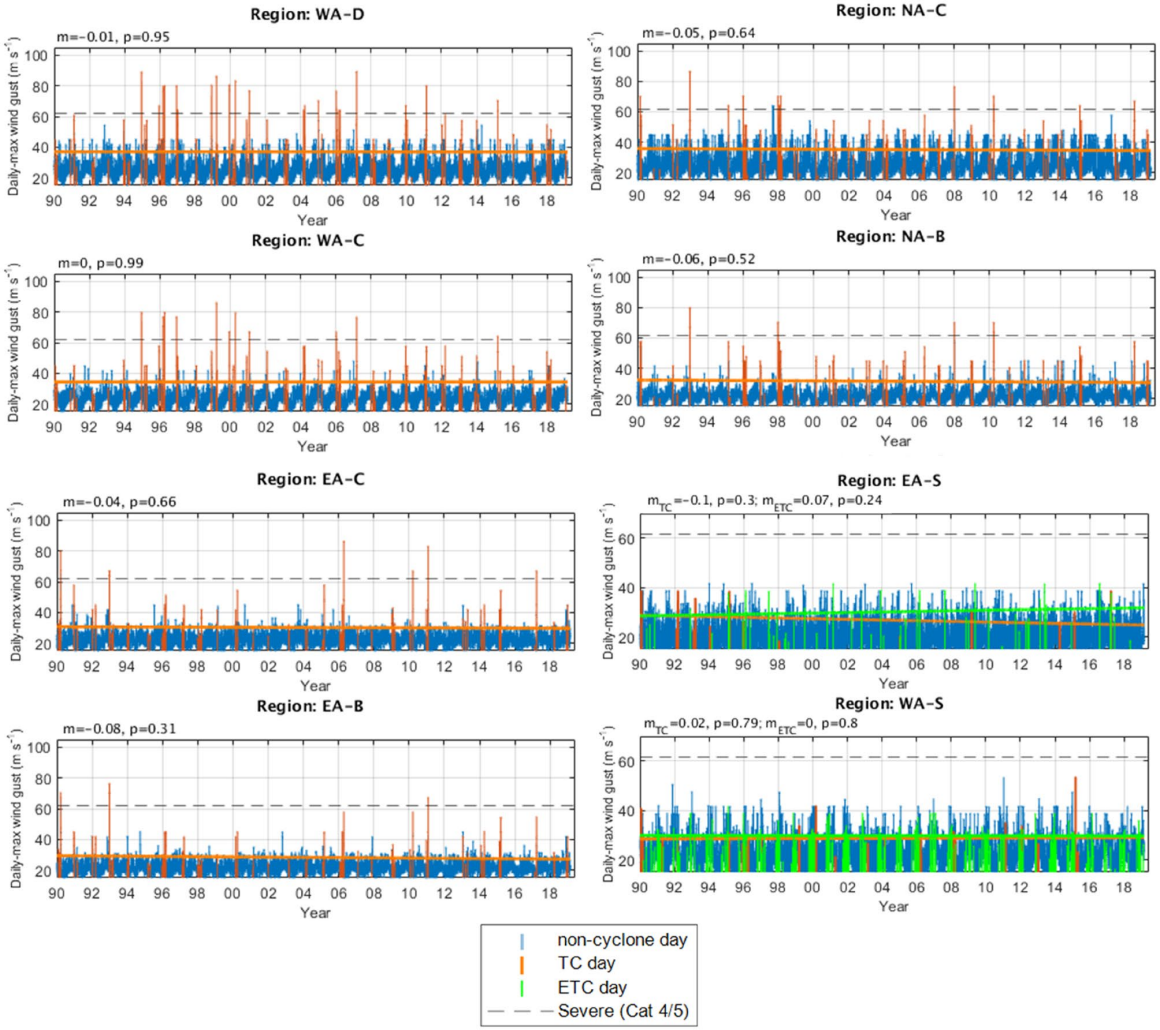
Time series and trend lines of calibrated BARRA reanalysis daily maximum wind gusts (3-s) occurring in each region. Daily maximum wind gusts are classified as “TC-related”, “ETC-related”, or “non-cyclone related” (see legend). Linear trend lines are shown for TC-day max winds (orange) and ETC-day max winds (green, where applicable). The estimate of the gradient (*m*) and associated *p*-value (*p*) for these trend lines are shown above each time series. Note that the gradient are shown for years (rather than TC days) for interpretability.

**Figure 4:**
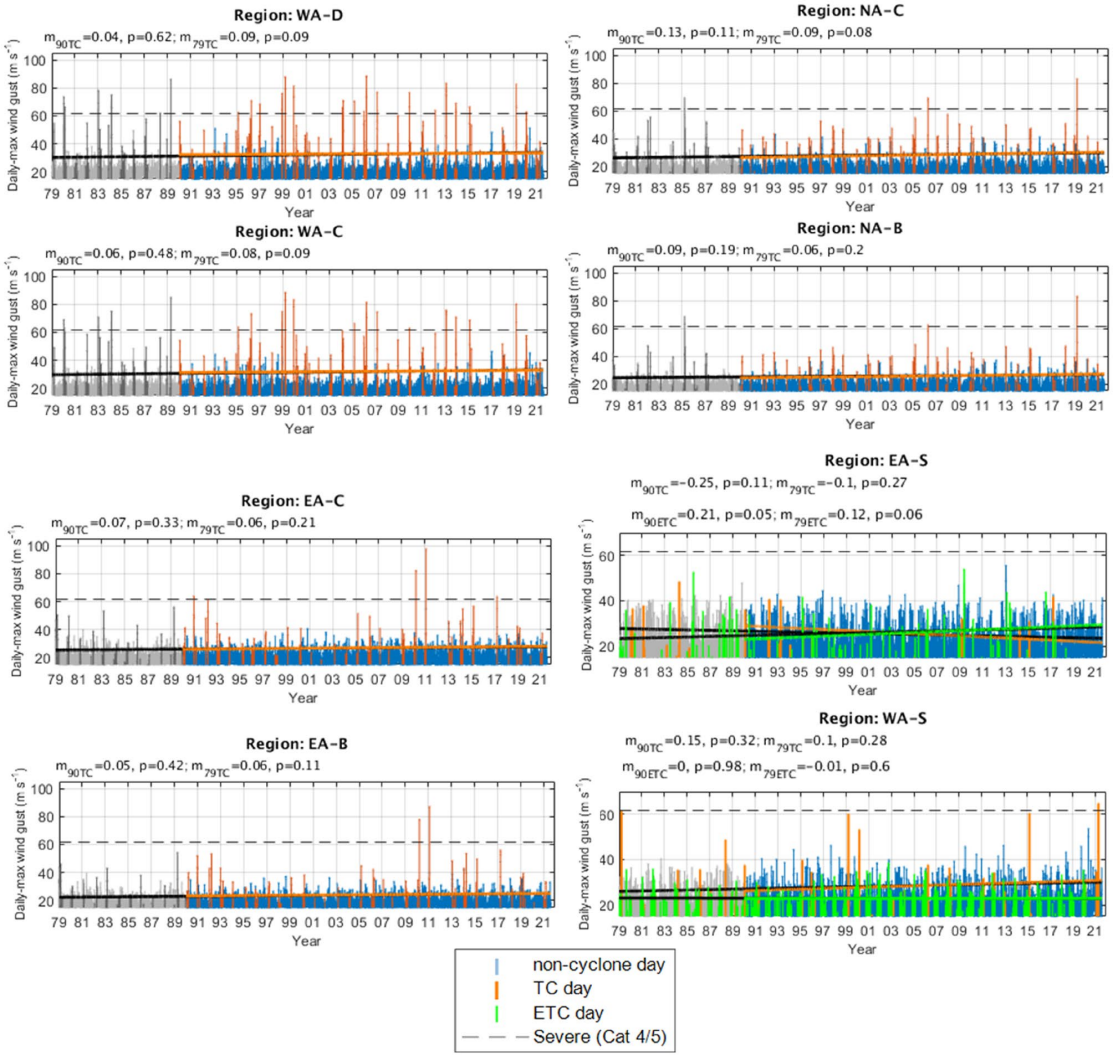
As in Fig. 3 but for the calibrated ERA5 reanalysis. As this data extends back to 1979, an additional trend line is fitted (black lines). Daily maximum wind gusts occurring before 1990 have been greyed out.

**Figure 5:**
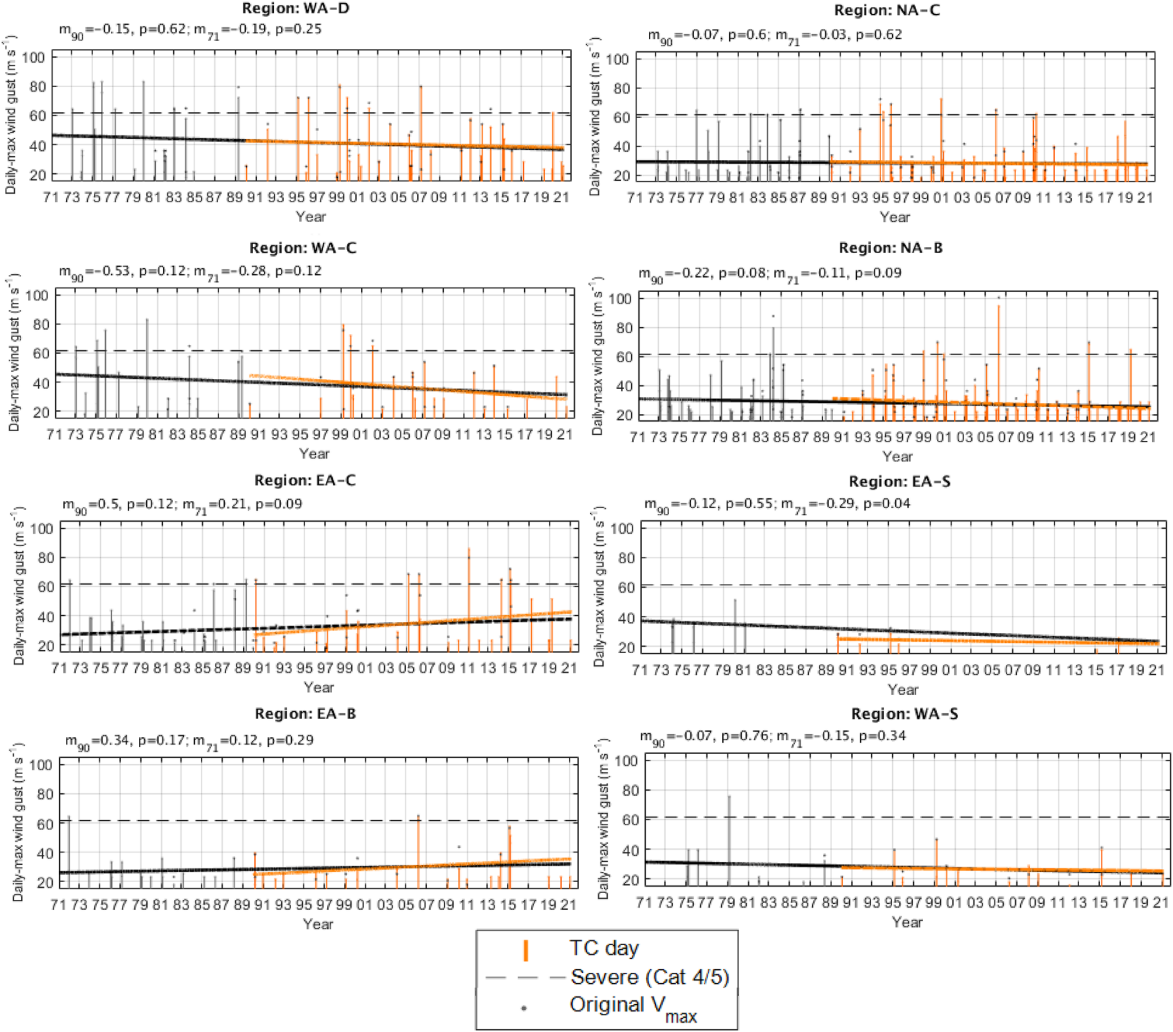
As in Fig. 3 but for best track TC wind gust speeds [replaced with adjusted V_max_ over the period 1981–2016 (Courtney and Burton 2018)].

Time series of daily-maximum wind gusts (3-s) related to TCs, ETCs (for the two subtropical regions) and “non-cyclones” (i.e., neither related to a TC or ETC) are shown for three datasets: BARRA (Fig. 3) ERA5 (Fig. 4) and Best track data (Fig. 5).

#### Tropical cyclone days

Linear trend lines are fitted to each time series of TC day maximum wind gusts (Figs. 3, 4, 5) and none are found to be significant at the 95% confidence level (i.e., *p* < 0.05), with exception of best track data back to 1971 in the EA-S region (Fig. 5). However, the limited number of samples and large differences between trendlines beginning from different start points (1990 and 1971), embeds a lot of doubt in this result.

Collectively, there are also differences in sign between at least one of the three datasets in each region. For example, if we consider that the different datasets provide a confidence range of the trendline coefficients for the combined tropical regions since 1990, these can be interpretated as (with excessively large annual trends from best track data shown separately with an asterisk (*):

- TC day maximum wind gusts on the Western Australian coast are insignificantly changing at a rate of − 0.5^*^ [− 0.01 to 0.06] m s^−1^ per year.
- TC day maximum wind gusts on the Northern Australian coast are insignificantly changing at a rate of − 0.2^*^ [− 0.06 to 0.1] m s^−1^ per year.
- TC day maximum wind gusts on the Eastern Australian coast are insignificantly changing at a rate of [− 0.08 to 0.07] 0.5^*^ m s^−1^ per year.

For the two subtropical regions since 1990:

- TC day maximum wind gusts affecting the EA-S region have a consistent downtrend between datasets but are insignificantly changing at a rate of [− 0.25 to − 0.1] m s^−1^ per year.
- TC day maximum wind gusts affecting the WA-S region are insignificantly changing at a rate of [− 0.07 to 0.15] m s^−1^ per year.

When considering trendlines back to the 1970s (i.e., from ERA5 and best track data; Figs. 4, 5), the trendline coefficients often become smaller, with exception of the tropical West coast regions and the subtropical regions for best track data (where trendline coefficients often become larger in size).

It is also practical to consider trends in the more extreme wind gusts that are most likely to cause significant property damage (i.e., Category 3 or above; > 45 ms^−1^). Trendlines for these “extreme” TC wind gusts (not shown) in combined regions since 1990 from the two reanalysis datasets can be interpreted as:

- TC day maximum wind gusts on the Western Australian coast are in some cases significantly decreasing at a rate of [− 0.53 to 0.06] m s^−1^ per year.
- TC day maximum wind gusts on the Northern Australian coast are insignificantly changing at a rate of [− 0.22 to 0.14] m s^−1^ per year.
- TC day maximum wind gusts on the Eastern Australian coast are insignificantly changing at a rate of [− 0.25 to 0.12] m s^−1^ per year.

#### Extratropical cyclone days

Considering the ETC day trend lines that are fitted to the two subtropical regions (BARRA and ERA5 only; Figs. 3, 4) the only significant result at the 95% confidence level (i.e., *p* < 0.05) was that of ETC wind gusts affecting the EA-S region since 1990 in ERA5: indicating a rate of increase of 0.2 m s^−1^ per year. This result was consistent in sign but was much greater in size when compared with BARRA and additionally ERA5 data back to 1979. There was no trend in ETC wind gust strength affecting the WA-S region.

For completeness, since 1990:

- ETC day maximum wind gusts affecting EA-S are in one case significantly changing at a rate of [0.1–0.2] m s^−1^ per year.
- ETC day maximum wind gusts affecting WA-S are insignificantly changing at a rate of [0–0] m s^−1^ per year.

### ARIs

**Figure 6:**
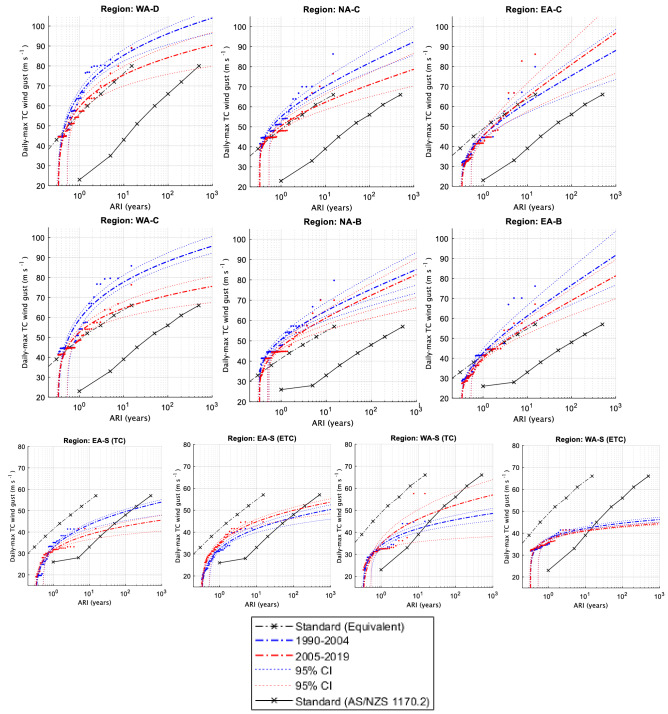
Average recurrence intervals (ARIs) of TC-related daily maximum wind gusts (0.2 s) for the calibrated BARRA reanalysis.

**Figure 7:**
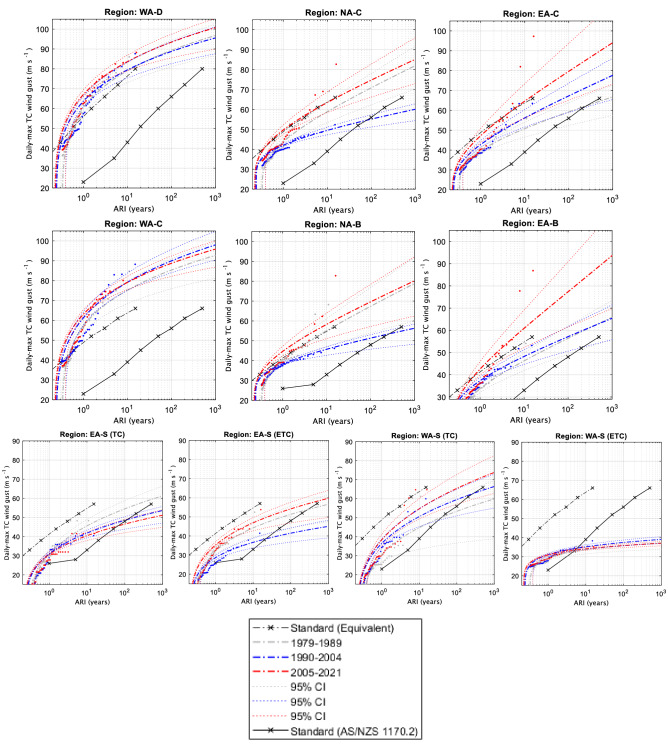
As in Fig. 6 but for the calibrated ERA5 reanalysis.

**Figure 8:**
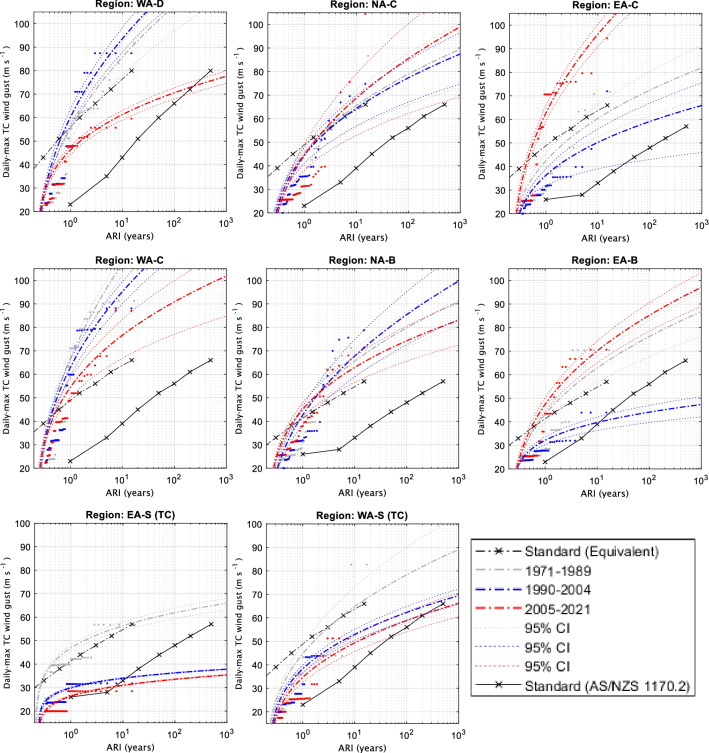
As in Fig. 6 but for best track TC wind gust data.

For comparison with the Standard wind curves, observed and model data are converted to an averaging period of 0.2 s gusts. However, this is still not a fair comparison as the Standard curves are extrapolations based on hundreds of years of data at several point locations inside a region while the reanalysis and best track curves are based on fewer years of data but at every available location (i.e., every grid cell in a region). Additionally, the reanalysis data have been calibrated to represent absolute wind speeds up to Category 5. So, it is expected that the curves based on the calibrated reanalyses (Figs. 6, 7) and best track data (Fig. 8) would have higher returns for small-to-medium ARIs and then approach the Standard when ARI is large (> 500). In an attempt to provide a fairer comparison between our ARI curves and the Standard ARI curves, we divide the Standard ARI curves by 33 (i.e., 500-year ARIs become 15-year and so on, under the assumption that 500 years of several point location wind speeds and 15 years of calibrated gridded wind speed data are each sufficiently long to sample the upper tail of winds expected in that region as a whole) to get equivalent curves for comparison which are additionally displayed to the regular Standard ARIs (dashed and solid black curves in Figs. 6, 7, 8).

#### Tropical regions

For the regions nearest to coastlines (WA-D, NA-C and EA-C; top rows in Figs. 6, 7, 8) the equivalent Standard curve generally lies in between or within the confidence estimates of the reanalysis and best track derived curves. However, this was not the case for the farther inland regions (WA-C, NA-B and EA-B; middle rows in Figs. 6, 7, 8) where the equivalent Standard curves were generally below the confidence estimates of the reanalysis and best track derived curves. This indicates that the reanalysis wind gusts did not decrease as would be expected farther inland and as compared to the Standard in some cases, particularly in the coarser ERA5 data (Fig. 7).

For the West coast regions (WA-D and WA-C), there is no clear change in the ARI time-slices covering 1990–2004 and 2005–2021 for ERA5 data (Fig. 7). However, BARRA (Fig. 6) and the adjusted best track record (Fig. 8) both showed some evidence of a slight downward trend in TC wind gust strength, including significant changes occurring in both these datasets. For the best-track record, the downward trend was consistent with the pre-1990 period, as these TC-gusts were similar in strength to the 1990–2004 period.

For the North coast regions (NA-C and NA-B), changes in the ERA5 ARI time slices (Fig. 7) depend on how much legitimacy is given to the pre-1990 data, as the first (1979–1989) and third (2005–2021) time-slices were nearly identical in terms of their respective ARI curves. If the pre-1990 data is not considered, there is evidence of an upward trend in ERA5 wind gust strength [i.e., a significant increase of ~ 20 m s^−1^ based on an ARI of 100] here in recent years, although this is much more likely related to decadal trends. Overall, BARRA showed that a slight downward trend in wind gust strength (~ 10 m s^−1^ for an ARI of 100 years) may have occurred over this same time period, although the BARRA results were not significantly different between the two time-slices and became negligible when considering the farther inland region (NA-B). The best track results were once again more similar to BARRA than ERA5, although no significant trends in wind gust strength were noted between the two most recent ARI time-slices (Fig. 8). For best track data, the extended pre 1990 data more closely resembled the 1990–2004 period (at least for NA-C) in terms of TC wind gust strength. In contrast, the pre-1990 ERA5 gusts more closely resembled the later ERA5 period (2005–2021) TC-gust strength (Figs. 7, 8).

For the East coast regions (EA-C and EA-B), a slight upward trend, significant for EA-B, between the two later ARI time slices is noted based on the ERA5 data (Fig. 7). It is interesting to note that the two eastern regions are the only cases where the ERA5 and best-track data seem to agree on the historical trends in TC wind gust strength, showing quite similar representations of each time-slice. BARRA also indicated a slight increase in TC wind gust strength over the most recent time-slice for the EA-C region (~ 5 m s^-1^ for an ARI of 100 years; Fig. 6), though this was not significant. Curiously, the reverse was true for the EA-B region (Fig. 6). This could potentially indicate that over the 1990–2004 period, winds maintained their strength farther inland than in the later period, though this was not noted in the best track data and could be due to a number of factors, including uncertainty in the BARRA data.

#### Subtropical regions

For the two subtropical regions (denoted with the suffix “-S” in Fig. 1), ARIs are shown in the lower panels of Figs. 6, 7, 8.

EA-S, is the only region analyzed in this study that has not been designated an additional climate change multiplier in the latest AS/NZS Standard^1^. The analysis undertaken in this study appears to support that decision as both the TC and ETC curves from all datasets are well below the equivalent Standard curves (Figs. 6, 7, 8, bottom row). Results between all three datasets are complementary in suggesting a potential small insignificant decrease for TC wind strength (Figs. 6, 7, 8); while the two reanalysis datasets are complementary in suggesting a small insignificant increase for ETC wind strength (Figs. 6, 7).

For the WA-S region, the TC curves also occur below the equivalent Standard curve with exception of the under-sampled pre-1990 best track curve (that is inconsistent with pre-1990 ERA5). Results between the two reanalysis datasets are complementary in suggesting a small but insignificant increase in wind strength (Figs. 6, 7), although this was not the case for best track data (Fig. 8). Results for the ETC curves indicated little change and occurred comfortably below the design Standard (even below that of a “B” designation which a majority of the WA-S region poleward of 27°S is currently designated).

## Discussion

Wind speed ARIs determined from TC climate data in two reanalysis datasets, and best-track observations for context, were compared to AS/NZS wind Standard curves^1^. Results indicated moderate reproduction of the ARI metric with both reanalysis datasets, although comparisons deteriorated when analyzing wind speeds farther inland than 50 km. The finer resolution BARRA product better simulated the Standard’s drop in TC-wind speed farther inland as compared to ERA5, an expected result. Differences in results between the three datasets highlight how important it is to consider multiple lines of evidence in these types of applications, especially with an eye to similar analyses using climate model simulations for future projections.

Overall, in terms of providing new lines of evidence in relation to historical TC-wind gust strength experienced in coastal regions of Australia in recent decades, analysis here of multiple datasets provides some evidence of a likely (2/3 datasets) slight downtrend in observed and estimated tropical West coast TC-wind gust strength with low-to-medium confidence, consistent with a prior study^5^. Similarly, there is some evidence of a likely (2/3 datasets) slight uptrend in the East coast TC-wind gust data with low-to-medium confidence consistent with other studies^4,30^. Noting however it may be unrealistic to see actual trends related to warming over such a short time period. There was low consistency in results between different data sets on the existence of trends in TC-wind gust strength along the North coast of Australia.

A significant trend in ETC-wind gust strength was found only for the subtropical East coast, where there was a small uptrend, consistent between both reanalysis datasets. West coast ETC wind gust strength was well below the current wind Standard, while East coast ETCs (East coast lows) were comparable, in terms of wind hazard, to the wind gust strength of TCs.

Overall, it is difficult to attribute any changes in extreme wind gust strength in these datasets to anthropogenic climate change at this time, given the relatively short time period and limitations of the data quality including over longer time periods and prior to the era of widespread satellite coverage in this region. More confidence in attributing such changes to greenhouse warming could be achieved by applying similar methods to fine (regional scale) and very fine resolution (convection-permitting) climate model experiments under atmospheric forcing. While observations are often favored when evaluating trends, climate models have the strong advantage of having multiple ensemble members, meaning that physically driven changes might be discernible well before a single realization in the real world is detected. Indeed, work in this respect is underway by these authors on applying this method to several regional climate model (RCM) simulations that are of similar resolution or finer to the reanalysis data used here (e.g., a new version of BARRA is being produced that will downscale from ERA5 reanalysis at its host model, then downscale further again to convection-permitting scales). The work done here has spelt out limitations in what we would be expected to be encountered by similar application with climate model data. In addition, similar analysis focusing on identifying a potential evolution in extreme rainfall hazards (including that induced by cyclones) could also be highly beneficial and important for managing future climate risk in Australia.

## Data Availability

The best track tropical cyclone dataset as well as all data related to the ERA5 reanalysis are freely available online. The BARRA data is located on NCI Australia and is available to researchers on reasonable request to Andrew Dowdy (Andrew.dowdy@bom.gov.au). For data specific to this study (e.g., TC wind gust time series for different datasets), please contact Samuel Bell (Samuel.bell@bom.gov.au).
